# Gene Expression Patterns of Hemizygous and Heterozygous *KIT* Mutations Suggest Distinct Oncogenic Pathways: A Study in NIH3T3 Cell Lines and GIST Samples

**DOI:** 10.1371/journal.pone.0061103

**Published:** 2013-04-12

**Authors:** Jean-Baptiste Bachet, Séverine Tabone-Eglinger, Sophie Dessaux, Anthony Besse, Sabrina Brahimi-Adouane, Jean-François Emile, Jean-Yves Blay, Laurent Alberti

**Affiliations:** 1 EA4340 ‘Epidémiologie et Oncogénèse des tumeurs digestives’, Faculté de médecine PIFO, UVSQ, Guyancourt, France; 2 Hepato-Gastroenterology Department, Groupe Hospitalier Pitié Salpêtrière, APHP, Paris, France; 3 Faculté de médecine Pierre et Marie Curie, UFR Paris VI, Paris, France; 4 Université de Lyon, Lyon, France; 5 Pathology Department, Hôpital Ambroise Paré, APHP, Boulogne, France; 6 Oncology Department, Centre Léon Bérard, Lyon, France; 7 Biopathology Department Centre Léon Bérard, Lyon, France; 8 Université Lyon 1, ISPB, Lyon, France; 9 INSERM U1052, Centre de Recherche en Cancérologie de Lyon, Lyon, France; 10 CNRS UMR5286, Centre de Recherche en Cancérologie de Lyon, Lyon, France; Virginia Commonwealth University, United States of America

## Abstract

**Objective:**

Most gain of function mutations of tyrosine kinase receptors in human tumours are hemizygous. Gastrointestinal stromal tumours (GIST) with homozygous mutations have a worse prognosis. We aimed to identify genes differentially regulated by hemizygous and heterozygous *KIT* mutations.

**Materials and Methods:**

Expression of 94 genes and 384 miRNA was analysed with low density arrays in five NIH3T3 cell lines expressing the full-length human *KIT* cDNA wild-type (WT), hemizygous *KIT* mutation with del557-558 (D6) or del564-581 (D54) and heterozygous WT/D6 or WT/D54. Expression of 5 of these genes and 384 miRNA was then analysed in GISTs samples.

**Results:**

Unsupervised and supervised hierarchical clustering of the mRNA and miRNA profiles showed that heterozygous mutants clustered with KIT WT expressing cells while hemizygous mutants were distinct. Among hemizygous cells, D6 and D54 expressing cells clustered separately. Most deregulated genes have been reported as potentially implicated in cancer and severals, as ANXA8 and FBN1, are highlighted by both, mRNA and miRNA analyses. MiRNA and mRNA analyses in GISTs samples confirmed that their expressions varied according to the mutation of the alleles. Interestingly, RGS16, a membrane protein of the regulator of G protein family, correlate with the subcellular localization of KIT mutants and might be responsible for regulation of the PI3K/AKT signalling pathway.

**Conclusion:**

Patterns of mRNA and miRNA expression in cells and tumours depend on heterozygous/hemizygous status of *KIT* mutations, and deletion/presence of TYR568 & TYR570 residues. Thus each mutation of *KIT* may drive specific oncogenic pathways.

## Introduction

Gastrointestinal stromal tumors (GISTs) are the most frequent sarcomas [1], and are thought to be derived from intestinal cells of Cajal or precursors. Gain of function mutations of proto-oncogenes *KIT* or *platelet-derived growth factor receptor alpha* (*PDGFRA*) play a critical role in GIST pathogenesis [2], [3]. *KIT* mutations are found in 85% of GISTs, and 5–10% for *PDGFRA* mutations; both are mutually exclusive [4]. Most of the *KIT* mutations are within the exon 11 (60%) with more than 90 different mutations described [4], [5], [6], [7]. Among them, the most frequent one is a short deletion in the proximal part of exon 11, delWK557-558, accounting for 8% to 25% of *KIT* exon 11 mutations [5]. Imatinib mesylate (Glivec®, Gleevec®, Novartis, Basel), a KIT and PDGFRA tyrosine kinase inhibitor, is the first-line reference treatment in advanced GISTs and in adjuvant setting [8], [9], [10]. The mutational status of *KIT* or *PDGFRA* is highly predictive of clinical response to imatinib, and patients with *KIT* exon 11 mutations have a significant longer progression free survival and overall survival than patients with *KIT* exon 9 mutations or wild-type GIST [11], [12]. Most of the GISTs have heterozygous mutations but homozygous mutations have also been reported accounting from 5% to 15% and seems to be associated with a worse outcome [13], [14], [15].

The *KIT* gene is a type III receptor tyrosine kinase whose activation follows the binding of its specific ligand, the stem cell factor (SCF). Kinase activation of KIT results in a cascade of phosphorylation promoting cell growth and survival [16]. Interestingly, *KIT* exon 11 encodes an intra-cytoplasmic juxtamembrane domain which has an autoinhibitory function [17]. More particularly, two tyrosine residues (Tyr568 and Tyr570) in the juxtamembrane segment are the first to be phosphorylated and are implicated in activation of different signaling pathways as of the Src family kinases [18].

In GISTs, as in cellular models, normal trafficking of the KIT protein results in the predominant expression of the fully glycosylated 145 kDa form, which is expressed at cell surface. The 125 kDa precursor form is also observed at a weaker level and it is retained in the intracellular compartment due to its incomplete glycosylation [19], [20]. Extracellular binding of the SCF induces phosphorylation of the mature form. In contrast, *KIT* activating mutations are associated with a constitutive phosphorylation of the immature form in the intracellular compartment [19], [20]. However distinct signaling pathways were recently reported to be activated according to the subcellular location of heterozygous or homozygous KIT mutations [21]. While most of the patients have heterozygous mutations [6], [19], [20], [21], [22], we have developed one of the rare model of GIST with NIH3T3 cell lines containing heterozygous mutations. We took advantage of this model to perform a large scale analysis of GISTs signaling pathways according to allelic status of two of the most frequent exon 11 mutations.

In the present work, we combined several high throughput analyses on hemi- or heterozygous *KIT* mutated cell lines as well as on GISTs samples. We demonstrated that the status of zygosity as well as the type of exon 11 mutation, in cell lines or in GISTs samples, possessed distinct gene expression and miRNA profiles. Interestingly, the heterozygous cell lines clustered more likely with wild-type cells than with hemizygous ones, thus decreasing importance of the mutated allele when coexpressed with the wild-type.

## Materials and Methods

### Reagents and Antibodies

Recombinant human SCF (rhSCF) used for the assays was purchased from R&D Systems and CHO-SCF, used for the culture, was a kind gift from P. Dubreuil. Imatinib mesylate was kindly provided from Novartis. The rabbit polyclonal anti-KIT antibody (clone A4502) was used for western blot, and the phycoerythrin-conjugated mouse monoclonal anti-KIT antibody (clone1045D2) for flow cytometry analysis (both from Dako, Trappes, France). The anti-phosphotyrosine 703 of KIT, used for western-blot and immunocytofluorescence analysis, were purchased from Clinisciences (Montrouge, France). Secondary-HRP antibodies (Dako) were used in western-blotting experiments.

### Production of Recombinant Viruses and NIH3T3 Infection

NIH3T3 cell lines (ATCC N°CRL-1658) expressing the full-length human *KIT* cDNA (GNNK isoform) wild-type (WT) or with either homozygous mutations (del557-558 = D6, or del564-581 = D54 mutation), or heterozygous mutations (WT/D6 or WT/D54) were previously described [19], [21]. Briefly, WT or mutated *KIT* were inserted in oncological vector pLNCX (Clontech®) and MigR1, respectively [23]; both retroviral vectors including the same cytomegalovirus promoter.

The plasmids containing WT and/or mutated *KIT* were transfected in 293T cells. The media containing virus was then harvested and used to infect NIH3T3 cells (multiplicity of infection of 0.5). NIH3T3 cells with the pLCNX vector were selected with geneticin for 7 to 10 days before being isolated and amplified. NIH3T3 cells with the MIGR1 vector were amplified for 48 h after infection and green fluorescent protein-positive cells were sorted.

A NIH3T3 cell line expressing the empty MigR1 vector was produce as control and was called MIGR.

### Cell Culture and Proliferation Study

NIH3T3-infected cells were cultivated in DMEM with 10% newborn calf serum (Life Technologies®), 2% penicillin/streptomycin, and 50 ng/ml of CHO-SCF so that the growths of wild-type and mutant forms were equivalent. CHO-SCF was removed during starvation performed before the assays. Recombinant human SCF (rhSCF) used for the assays was purchased from R&D Systems®.

### High Density Arrays (Codelink®)

Codelink® Whole Mouse Genome Bioarrays (GE/Amersham) of one sample of WT, D6, D54, WT/D6, WT/D54 and MIGR NIH3T3 cells cultivated in the three different conditions SCF+, SCF− and imatinib were used for the analysis of differential gene expression. These microarrays contain 3.3×10^4^ single-stranded 30-mer oligonucleotide probes for mouse genes and transcribed sequences.

For the experiment, the various NIH3T3 KIT expressing cell lines were seed at 1 million per 6 cm plate in complete medium without SCF and keep in this condition during one night (16 hours). Then, the cells were incubated for 24 hours with or without rhSCF and/or imatinib as indicated.

Before RNA extraction, cells were detached following a classical trypsine protocol (Invitrogen®), washed two times in cold PBS (Invitrogen®) and finally freezed at −80°C without supernatant.

Total RNA of NIH3T3 cells were extracted according to the manufacturer’s protocol using TRIzol® method (Invitrogen®, Carlsbad, CA). RNA quality was controlled by spectophotometry using ND-1000 Nanodrop® and by microchips on Agilent bioanalyzer 2100 (Agilent® Technologies, Palo Alto, CA, USA). Then, for high density microarrays, total RNA (100 ng) were amplified and biotin-labeled by in vitro transcription (IVT) with a MessageAmp™ II-Bacteria RNA Amplification Kit (Ambion/Applied Biosystem®). Before amplification, spikes of synthetic mRNA at different concentrations were added to all samples; these positive controls were used to ascertain the quality of the process.

Ten micrograms of biotinylated cRNA was fragmented and hybridized onto Codelink® Whole Mouse Genome Bioarrays (GE/Amersham) for 18 hours at 37°C at 300 rpm in an incubator. The slides were washed and stained with streptavidin-cy5 (GE Healthcare) according to the manufacturer’s protocol. The slides were scanned using a Genepix® 4000B scanner (Axon, Union City, USA) and Genepix® software, with the laser set at 635 mm, the laser power at 100%, and the photomultiplier tube voltage at 60%.

The scanned image files were analyzed using CodeLink® expression software, version 4.2 (GE Healthcare), which produces both a raw and normalized hybridization signal for each spot on the array. The relative intensity of the raw hybridization signal on arrays varies in different experiments. CodeLink® software was therefore used to normalize the raw hybridization signal on each array to the median of the array (median intensity is 1 after normalization) for better cross-array comparison. The threshold of detection was calculated using the normalized signal intensity of the 100 negative control samples in the array; spots with signal intensities below this threshold are referred to as “absent”. The complete set of raw and normalized data is available at the GEO database under accession number GSE33968.

Selections of genes were performed by comparisons between group1 and group2 or group1 and group3 … Each sample from one group was compared with each sample from others groups, and only genes showing a variation of average fold change ≥2 were retained. A gene was considered only if it met the above criteria in all comparisons and if the detected signal was above the background for at least one of the compared tumor groups, thereby carrying a statistically significant absolute call ‘present’ or ‘marginal’ in all samples. The retained genes of interest were listed and classified according to their biological functions on the basis of the Gene Ontology Consortium using Ingenuity® Pathway Analysis (Mountain View, CA).

### Taqman® Low Density Arrays Gene Expression

For low density arrays, RNA was extracted as for high density array. Then, 5 µg of RNA was submitted to reverse transcriptase using High-capacity cDNA Archive kit (Applied Biosystems®). As control, screening of WT and/or D6 or D54 *KIT* exon 11 was performed by length analysis of polymerase chain reaction products in all cell lines (MIGR, WT, D6, D54, WT/D6, WT/D54 NIH3T3) [24].

The 94 genes of interest and one stable mRNA (*MYH9*) were chosen after analysis of high density arrays. Microfluidic cards performing high-throughput Taqman®-based PCR assays, called Taqman® Low Density Arrays (TLDA), of these 95 selected genes were obtained from Applera®, Courtaboeuf. Each RNA sample was independently obtained after cells culture in specific condition, total RNA extraction with TRIzol®, and cDNA production. In condition SCF+, three complete replicates of WT, D6 and D54 NIH3T3, and two complete replicates of WT/D6 and WT/D54 NIH3T3 were obtained. In condition imatinib (rhSCF +1 µM imatinib), two complete replicates of WT, D6, D54, WT/D6 and WT/D54 NIH3T3 were produced. For MIGR NIH3T3, two complete replicates in condition SCF+, SCF− and imatinib were obtained. Real-time quantitative PCR on TLDA were done with the Abi Prism® 7900HT sequence detection system.

### Analysis of miRNA Using TLDA

Total RNA was extracted from pelleted cell lines using TRIzol® method, according to the manufacturer’s instructions (Invitrogen®, Carlsbad, CA). 500 ng of total RNA were subjected to the microfluidic PCR technology performed by Applied® Biosystems. We obtained cDNAs complementary to 384 identified miRNAs using a pool of looped miRNA-specific RT primers, according to the Megaplex® RT reaction protocol. PCR quantification was performed using a TaqMan® MicroRNA Array (TLDA human pool A array) allowing the independent amplification of these 384 miRNA. Q-PCR was performed using a 7900 Sequence Detection System, which calculated the threshold cycle, or Ct (plate by plate manual Ct analysis with a threshold at 0.25, and automatic baseline).

### TDLA Data Normalisation, mRNA and miRNA Analyses

For mRNA analysis, results, expressed as N-fold differences in target gene expression relative to the normalization gene, and termed "Ntarget", were determined as Ntarget = 2Δ^Ctsample^, where the Δ^Ct^ value of the sample was determined by subtracting the average Ct value of the target gene from the average Ct value of the normalization gene (plate by plate manual Ct analysis with a threshold at 0.25 and automatic baselines). We tested different methods of normalization since the “pseudo” stable *MYH9* gene plotted in each card was not stably expressed in our different samples. Normalisation with the four most stable mRNAs identified in GeNorm (*Smc1a, Usp33*, *Nsmaf* and *Rabep1*) was not optimal too. Finally, a global normalization by the median was chosen for its reliability over experiments. Distribution of normalized data was checked with box plots and correlation plots.

For each mRNA, the threshold cycle (Ct) was calculated with the RQ Manager 1.2 software (Applied Biosystems®). Data manipulations were done using R scripts and automated Excel sheets. A cut-off at 35 Ct was applied to discard late Ct values. For each TLDA, quality controls were performed on the raw data by checking internal controls and using box plot and scatter plot diagrams. Samples with any kind of problems were discarded so they would not introduce bias during the global normalization procedures. The following formula was used to correct Ct values of every card:
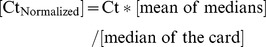



Through this approach, the new median value shared by all samples can be considered as a sort of perfect “virtual housekeeping gene”. Therefore the standard ΔΔCt method can be used to determine the relative quantities as follows:
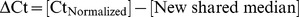



For the ΔΔCt calculation, as described previously [25], it was more relevant for the statistical analyses to use the mean of all ΔCt obtained across samples for each mRNA, instead of using the ΔCt of a reference sample:
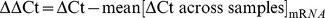


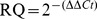



The same methodology was used for miRNA analysis.

For statistical analysis, normalized relative quantities data were directly input into the TIBCO Spotfire® DecisionSite for Functional Genomics analysis software. Data of interest were then used as variables in a two dimensional principal component analysis (PCA) performed with R 2.9.0 package to demonstrate their capabilities to distinguish the different cell lines. PCA supplies a simplified two-dimensional picture to our multivariate dataset of mRNA RQ values. By mathematical combination of values according to their strength, two principal components are created that represent as much as possible the variability of the data.

### GISTs miRNA and mRNA Analyses

For mRNA analysis, we quantified 5 mRNA (*ANXA8*, *FBN1*, *GALNTL4*, *MFAP5* and *RABEP1)* of the highlighted genes in the gene expression analysis of our cell lines in 4 WT-GIST, 5 *PDGFRA* mutants and 7 *KIT* mutants. Samples were formalin-fixed paraffin-embedded tumors. 100 ng of total tumoral RNA were used for each single qPCR reaction. Reverse transcription was performed using the high capacity cDNA reverse transcription kit (Applied Biosystems®) according to the manufacturer’s instruction. To verify the efficacy of provided random primers, specific reverse transcription was simultaneously performed with gene’s specific primers at a final concentration of 0.5 µM. qPCR reactions were performed using custom 96-well plates TaqMan® Gene Expression Assay following the program of heating to 95°C for 10 minutes and 40 cycles of 15 seconds at 95°C and 1 minute at 60°C. Assays are summarized in table 1.

**Table 1:** mRNA analysis in GIST samples: details of selected genes.

Gene name	Assay reference	Specific primers sequences (5′ ->3′)
*18S*	Hs99999901_s1	GCCCGCTCCCAAGATCCAACTACGA
*ANXA8*	Hs00179940_m1	GCGATCTGCTGCCGCTGCGT
*ETV6*	Hs01045742_m1	AACTCATTTTCAGCCCACTT
*FBN1*	Hs00171191_m1	TGACCAGATGGGCAAGTGCA
*GALNTL4*	Hs00289325_m1	GCACTGCAGCTGTTTCCTGA
*MFAP5*	Hs00185803_m1	CGATGCACAGAGTAGAGCCT
*RABEP1*	Hs00388983_m1	GCTGCTGTTGTGCACGTAAA

Data normalization: A cut-off of 37 was applied to discard late Ct values. As the recommended *18S* normalization gene was found very variable in our experiments (standard deviation of 5.5 Ct), normalization was performed using the *ETV6* gene (standard deviation of 1.5 Ct) shown as stable on cell lines’ experiments. We calculated the mean Ct for ETV6 on all our experiments and used this value to determine tumor-specific correcting factors F, with i genes and j patients:
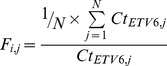



Ct values were then corrected for each tumor and ΔCt values were calculated as follows:
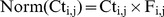


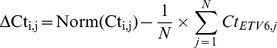



For miRNA analysis, 20 patients diagnosed with GIST were included in this study. Groups were formed regarding to the kind of mutations harbored, regardless to any other variable. However, as no homozygous *KIT* mutated sample was available in this first mRNA analysis, we performed a second analysis of miRNA profiling on a new serie of GISTs; all are mutated on the exon 11 with 6 of homozygous and 13 heterozygous. Groups were formed regarding to the homozygous or heterozygous status of exon 11 *KIT* mutation, regardless to any other variable. Both histological and molecular analyses were performed at the Centre Léon Bérard in Lyon. Samples were formalin-fixed paraffin-embedded tumors. For tumor fragments, two phenol/chloroform extractions were performed with a DNAse step in between. miRNA profiling was performed using the TLDA platform and the human 384-microfluidic card A. As described for miRNA analysis form cell lines, the same method was used for the ΔΔCt calculation and the statistical analysis [25].

### Statistical Analyzes

We performed univariate and multivariate logistic regression analyses to examine the relationship between the clinical data and the homozygous/heterozygous status. We studied multiple factors: age, gender, tumor size, mitosis, necrosis, risk of relapse, histology type, primitive location, kit immunohistochemistry and kit intracellular locations (Golgi, membrane, cytoplasm). Quantitative variables (age, tumor size, mitosis) were categorized. The different cutoffs used to create two groups for each factor were: 60 years (age), 6 cm (tumor size), 2 (mitosis). Furthermore, Fisher's exact test was used for the 2-categorical variables (age, gender, tumor size, mitosis, kit intracellular locations).

### Ethics Statement

Patients with GISTs of this study were included in the MolecGIST study which is a prospective population-based study of GISTs. Participants in MolecGIST provide their verbal informed consent to participate in this study after reading an information note.

Patients were informed that molecular analyses from the tumor would be conducted within the framework of the study. These analyzes relate only to the resected tumor sample in the context of routine care and no additional sample was collected in this study. In the absence of germline analysis on the patient himself, a written consent was not requested by the French ethical committee.

The process of information and oral consent was not documented, because it was not required by the ethical committee. Details concerning the study are publicly available on http://www.gist-france.org/tumeur.html#tumeur since April 6^th^ 2006.

MolecGIST was approved by French ethical committee: "Comité pour la Protection des Personnes se prêtant à des Recherches Biomédicales" (CPPRB, Committee for the Protection of Persons suitable for Biomedical Research) Saint Germain en Laye #06029, April 24th 2006.

Informed consent was obtained from all patients.

## Results

### High Density Arrays (Codelink®)

The gene expression profiles of the cell lines were first performed to a high density. The aim of this analysis was to identify the most relevant genes that characterized the different c-kit cell lines and then to validate them by TLDA analysis. From the complete set of raw (GEO database GSE33968), we selected genes differentially expressed between WT and hemizygous samples grown in the SCF+ condition, whose fold change was superior or equal to 2 and whose expression for at least one of the sample was greater than 1.3 (commonly admitted threshold for low density arrays). We then selected 83 and 126 genes more, that were differentially expressed between D6 and D54 cell lines (fold change ≥2; gene amplification >1.3), in SCF+ or SCF− condition, respectively. Finally we obtained a list of 235 genes of which 94 were available for Taqman® low density array (Applied®).

### TLDA and Gene Expression Profiles

**Figure 1:**
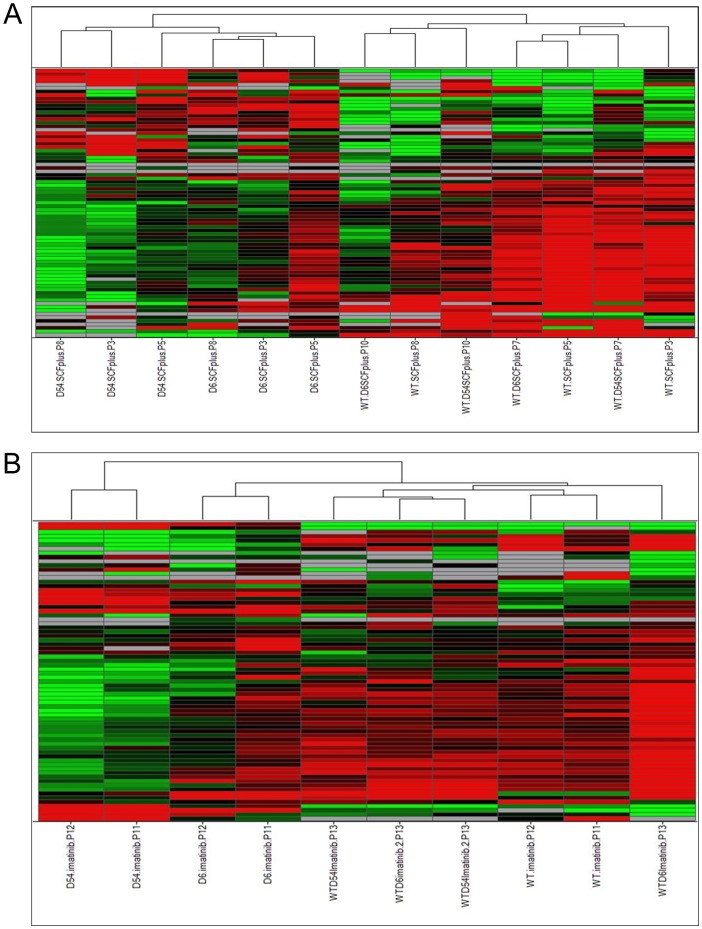
Unsupervised Hierarchical clustering of mRNA expressions in cell lines. A/upon SCF treatment, or B/with imatinib treatment. Each row represents the relative levels of expression of each mRNA and each columm shows the expression levels for each sample. The red or green color indicates relatively high or low expression, respectively, while grey squares indicate absent data points (WT, wild-type *KIT*; D6, D6 *KIT*; D54, D54 *KIT*; WT/D6, WT *KIT* and D6 *KIT*; WT/D54, WT *KIT* and D54 *KIT*).

The mRNA expression profiles of the various cell lines were performed using low density array on the 94 selected genes. Firstly, to eliminate possible non specific mRNA, we performed unsupervised hierarchical clustering of MIGR NIH3T3 samples cultivated in the three different conditions (SCF+, SCF−, imatinib). Using ANOVA tests with *p*-values of 0.05, mRNAs which were statistically differentially expressed in MIGR NIH3T3 samples were considered non specific of KIT pathways and were excluded for further analysis. Then, we performed unsupervised hierarchical clustering to classify WT, D6, D54, WT/D6, WT/D54 NIH3T3 samples by groups. The selection of mRNAs useful to differentiate cell line samples under SCF+ or imatinib conditions was statistically realized using ANOVA tests with *p*-values of 0.05 at least. Upon SCF treatment, an unsupervised hierarchical clustering based on Euclidian distance revealed that heterozygous mutants clustered together with WT cells, whereas hemizygous mutants were distinct from WT cells and heterozygous mutants (Figure 1A). In the presence of 1 µM imatinib, an unsupervised hierarchical clustering also shown that heterozygous mutants clustered together with WT cells. By contrast, D6 and D54 hemizygous mutants were clustered distinctly from each other and segregated also differently from heterozygous mutants and WT cells (Figure 1B).

**Figure 2:**
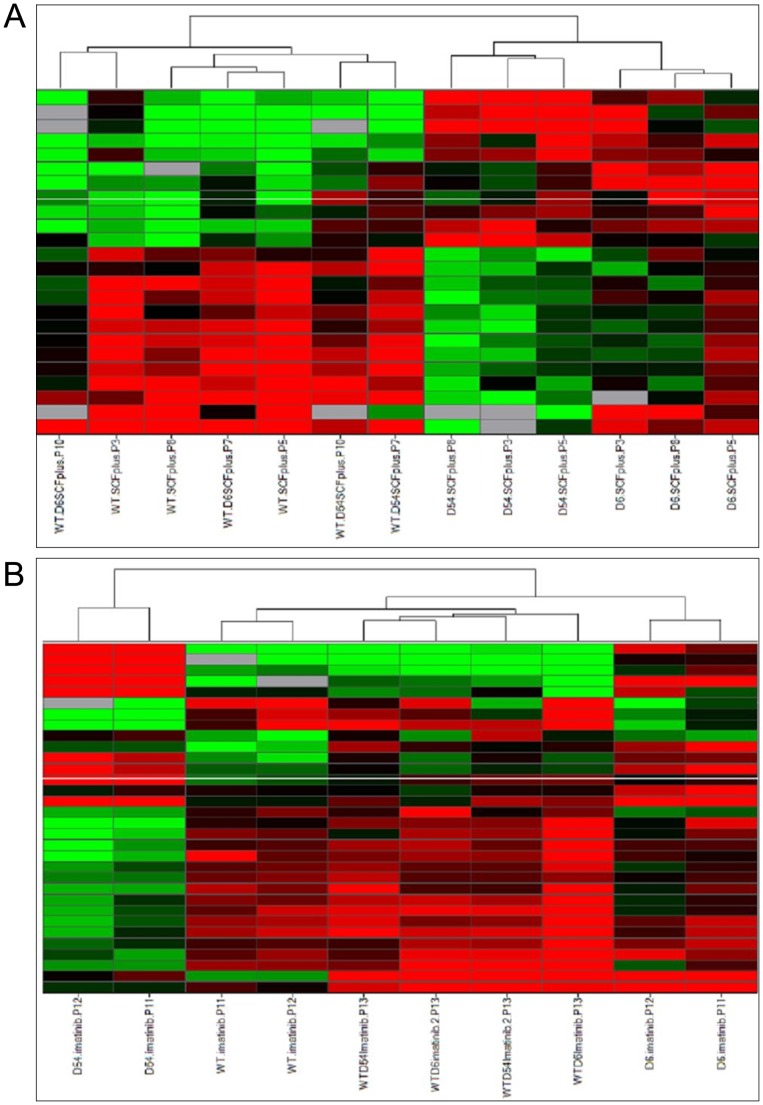
Supervised Hierarchical clustering of mRNA expressions in cell lines following an ANOVA. A/upon SCF treatment or B/with imatinib treatment. Each row represents the relative levels of expression of each mRNA and each columm shows the expression levels for each sample. The red or green color indicates relatively high or low expression, respectively, while grey squares indicate absent data points (WT, wild-type *KIT*; D6, D6 *KIT*; D54, D54 *KIT*; WT/D6, WT *KIT* and D6 *KIT*; WT/D54, WT *KIT* and D54 *KIT*).

Thereafter, we performed a supervised hierarchical clustering following an ANOVA (*p*<0.04) between the five transfected cell lines. The clustering confirmed proximity of WT and heterozygous mutants’ profiles upon SCF stimulation, while hemizygous mutants were still distinct (Figure 2A). Again, in the presence of 1 µM imatinib, hemizygous mutants were clustered separately and distinct from WT cells and heterozygous mutants, but heterozygous mutants were slightly distinct from WT cells (Figure 2B).

**Figure 3:**
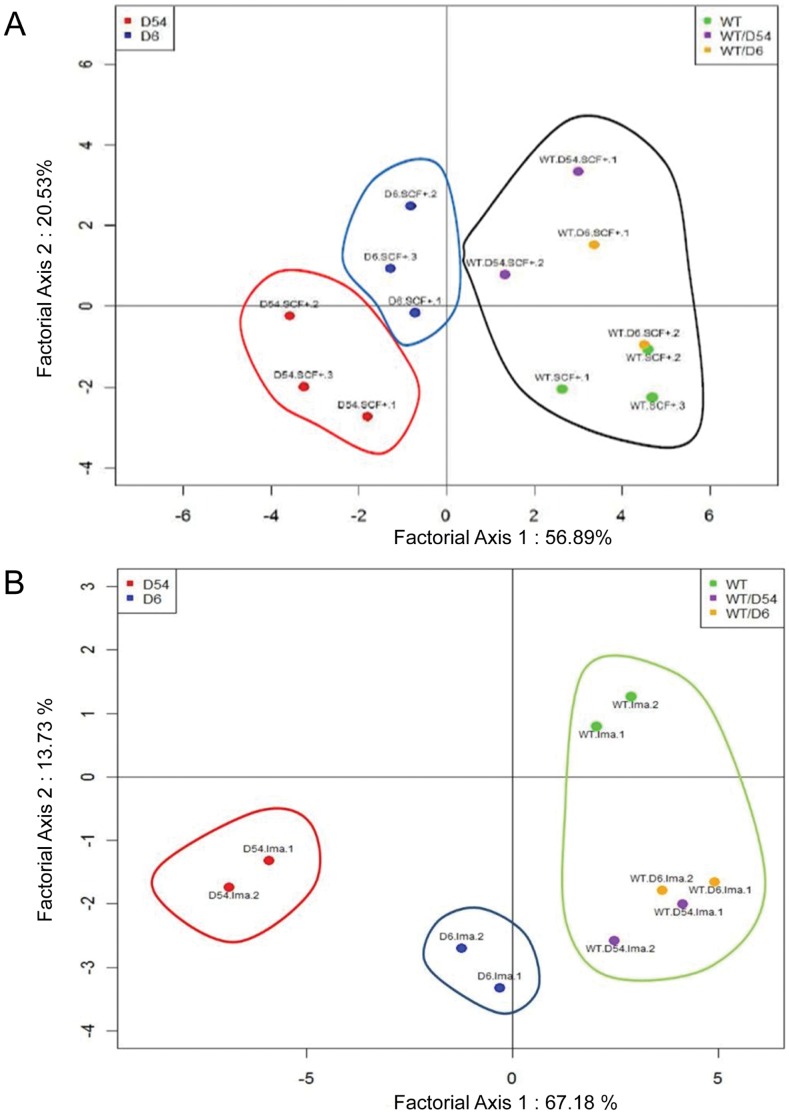
Principal component analysis (PCA) diagram of the 23 selected genes. A/upon SCF treatment or B/with imatinib treatment. PCA of mRNA selected genes in NIH3T3-infected cell lines as a tool to determine their capabilities to distinguish the different cell lines (WT, wild-type *KIT*; D6, D6 *KIT*; D54, D54 *KIT*; WT/D6, WT *KIT* and D6 *KIT*; WT/D54, WT *KIT* and D54 *KIT*).

Using a statistical analysis based on principal composant analysis, we selected 23 genes which were frequently deregulated between WT cells *vs* heterozygous *vs* hemizygous mutants: *Anxa8, Farp2, Fbn1, Galntl4, Gpr149, Ifi47, Mfap5, Ncam1, Nf1, Olfr30, Ptx3, Rabep1, Rgs16, Riok1, Runx1t1, Slit3, Thsb1, Trim66, Ulk1, Upp1, Usp33, Wisp1, Wisp2*. This mathematical procedure assigned for each contributory mRNA in transfected cell lines a new coordinate system, composed of two coordinates, which allow to study the variability between a set of variables. The results were consistent with those of unsupervised and supervised hierarchical clustering. In the SCF+ condition, heterozygous mutants and WT cells were localized in the same area whereas samples of each hemizygous mutant were independently pooled (Figure 3A). In the presence of imatinib, four different groups of cell lines were clearly individualized: WT cells, heterozygous mutants, D6 and D54 mutants (Figure 3B).

### miRNA Expression on KIT-expressing NIH3T3 Cells Classify Samples According to the Mutation Type and to the Presence of an Additional Wild-type Allele

We then performed miRNA profiling on the *KIT* expressing WT, D6, D54, WT/D6 and WT/D54 cell lines. We first eliminated unspecific miRNA by identifying deregulated miRNA upon transfection. Hence, 61 miRNAs were differentially expressed in NIH3T3 and MIGR cell lines (ANOVA test *p*<0.01). Among the 384 tested miRNA, 184 were deregulated by KIT expression in these cell lines. Finally, although a moderate significance of the ANOVA test between WT and mutant cell lines (p<0.07) due to the high background of NIH3T3 cell lines, 11 miRNA were specifically related to the presence of a hemizygous or heterozygous *KIT* mutation: miR-19b, miR-26a, miR-26b, miR-28-3p, miR-29a, miR-29b, miR-100, miR-192, miR-218, miR-222, and miR-708.

**Figure 4:**
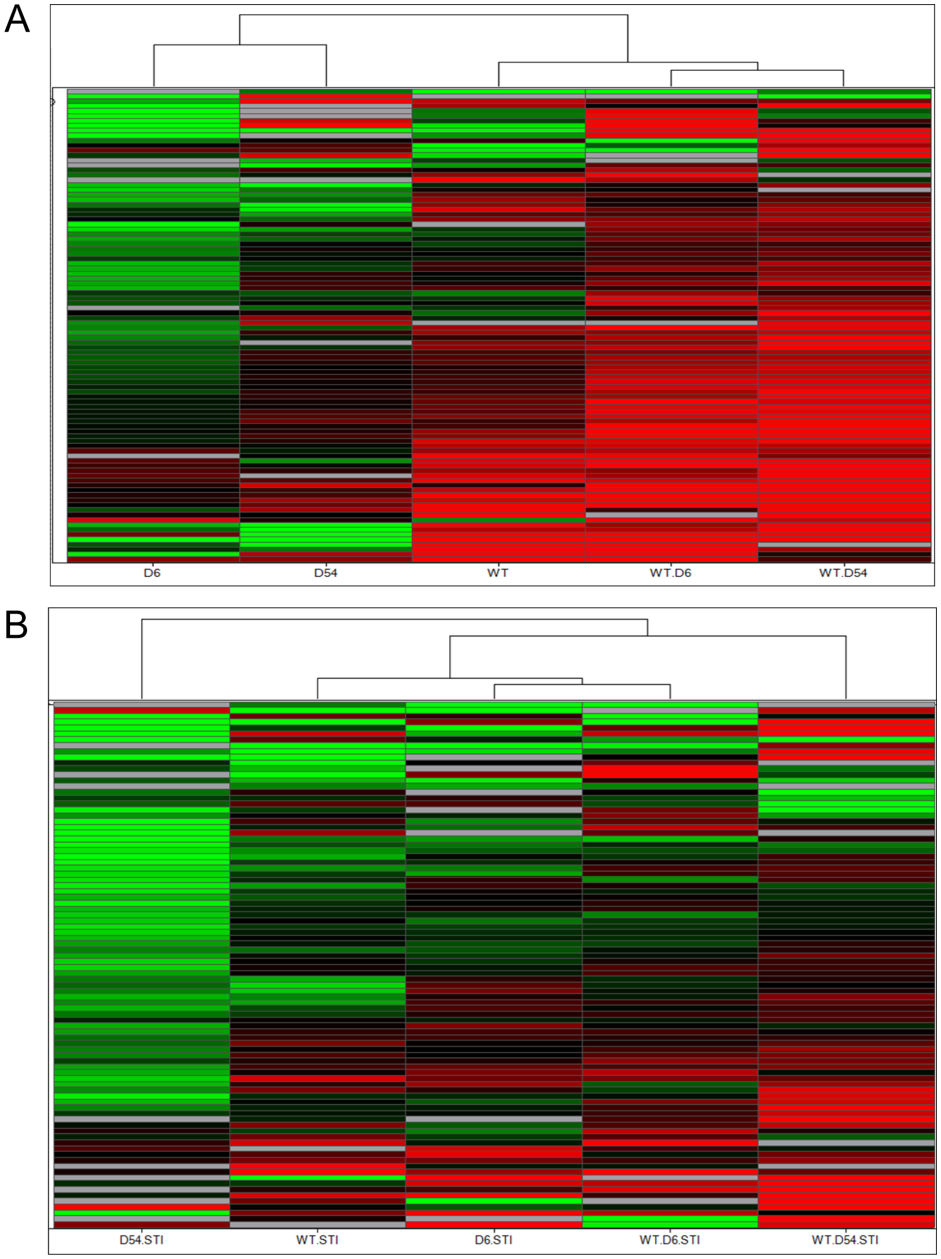
Unsupervised hierarchical clustering of miRNA expressions in cell lines. A/upon SCF treatment or B/with imatinib treatment. Each row represents the relative levels of expression of each miRNA and each columm shows the expression levels for each sample. The red or green color indicates relatively high or low expression, respectively, while grey squares indicate absent data points (WT, wild-type *KIT*; D6, D6 *KIT*; D54, D54 *KIT*; WT/D6, WT *KIT* and D6 *KIT*; WT/D54, WT *KIT* and D54 *KIT*).

An unsupervised hierarchical clustering revealed that upon SCF stimulation WT cells were branched to the heterozygous mutants group (Figure 4A). Hemizygous cell lines clustered separately, which is consistent with our previous gene expression analysis. Upon imatinib treatment, the miRNA profile of D6 cells get together with the WT/D6 mutant. WT cells were still branched to the heterozygous cell lines, while D54 cells remained separated (Figure 4B).

**Figure 5:**
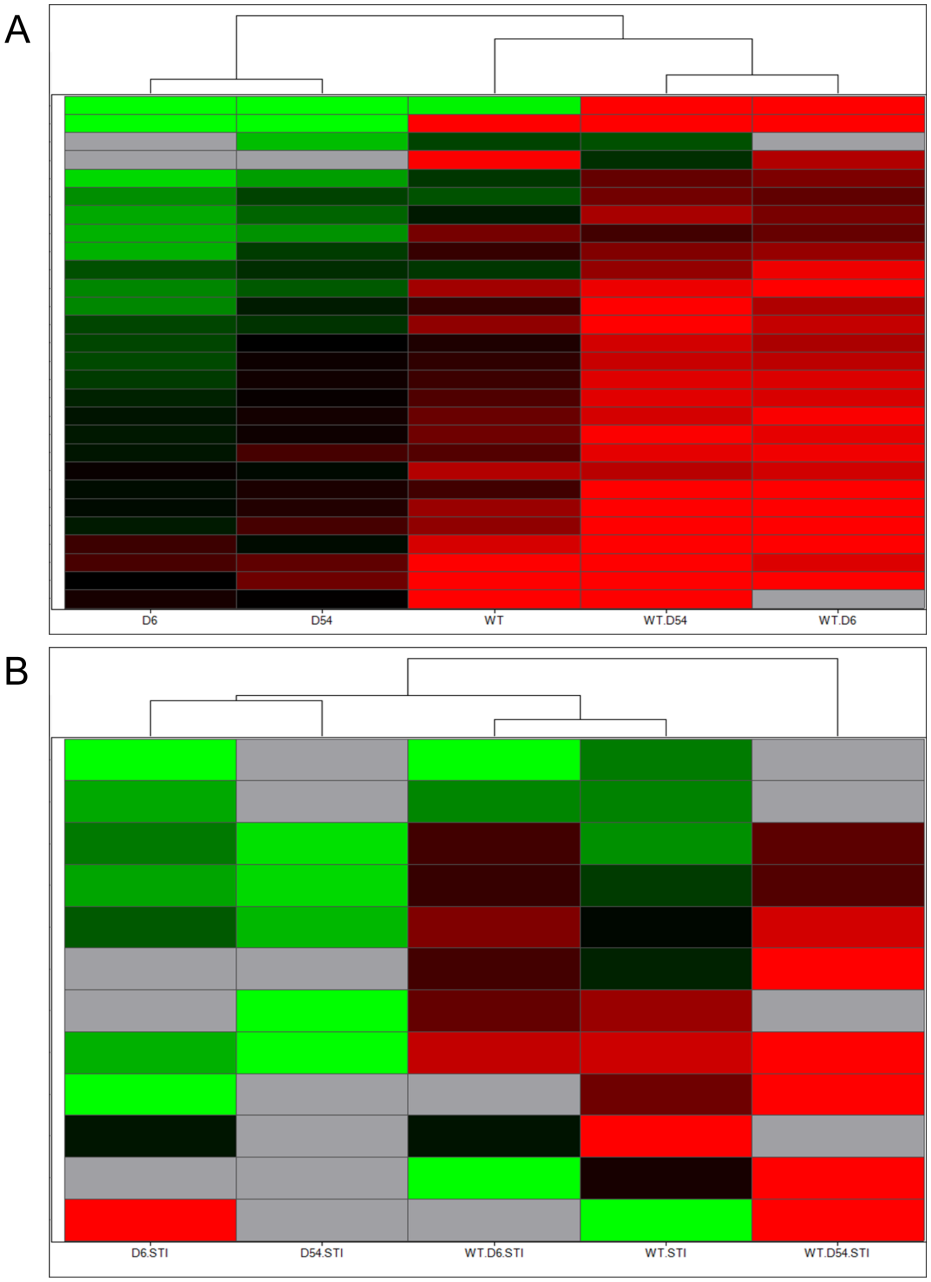
Supervised hierarchical clustering of miRNA expressions in cell lines following an ANOVA. A/upon SCF treatment or B/with imatinib treatment. Each row represents the relative levels of expression of each miRNA and each columm shows the expression levels for each sample. The red or green color indicates relatively high or low expression, respectively, while grey squares indicate absent data points (WT, wild-type *KIT*; D6, D6 *KIT*; D54, D54 *KIT*; WT/D6, WT *KIT* and D6 *KIT*; WT/D54, WT *KIT* and D54 *KIT*).

Supervised hierarchical clusterings were then performed using an ANOVA test at 5% between hemizygous and heterozygous cell lines. As a supervised analysis required groups, the WT cell line was added following the ANOVA test. We observed that untreated WT cells clustered with heterozygous cells while hemizygous cell lines clustered separately, leading to a clustering similar to Figure 5A. After imatinib treatment, hemizygous appeared as a define group (Figure 5B). WT and WT/D6 cells were enclosed together, while WT/D54 cells were separated.

### miRNA and mRNA Expression Patterns of GIST Classify Tumors Mainly According to their Mutational Status

To confirm the relevance of the molecular signatures found in our vitro model we analyzed the profiles of GIST samples whose clinical and molecular characteristics are detailed in Table 2.

**Table 2:** Characteristics of GISTs included in miRNA and mRNA analyses.

Tumor reference	Anatomic site	Risk ofrelapse*	Sex	Age(years)	*KIT* Exon 9 mutation	*KIT* Exon 11mutation	*PDGFRA* Exon 18 mutation	*PDGFRA* Exon 10 mutation
F1511^•▴^	rectum	high	M	70	WT	WT	WT	wt
F1521^•▴^	stomach	moderate	M	69	WT	WT	WT	−
F1522^•▴^	small intestine	moderate	M	42	WT	WT	WT	wt
F1529^▴^	small intestine	moderate	F	75	WT	WT	WT	−
F1507^•▴^	peritoneum	high	M	53	WT	WT	D842V	−
F1508^▴^	−	high	M	67	WT	WT	D842V	−
F1525^•▴^	peritoneum	high	F	69	WT	WT	D842V	−
F1526^•▴^	stomach	moderate	F	86	WT	WT	D842V	−
F1527^•^	−	−	F	71	WT	WT	D842V	−
F1528^•^	stomach	low	M	80	WT	WT	D842V	−
F1524^•▴^	stomach	low	M	70	WT	WT	WT	poly
F1535^•^	small intestine	high	M	67	mutated	WT	WT	−
F1537^•▴^	small intestine	moderate	F	69	mutated	WT	WT	−
F1538^•▴^	small intestine	low	M	83	mutated	WT	−	−
F1539^▴^	small intestine	low	F	65	mutated	WT	WT	−
F1540^▴^	mesentery	high	F	65	mutated	WT	WT	−
F1541^•▴^	−	−	M	80	mutated	WT	WT	−
F1550^•^	stomach	low	F	51	WT	W557R	WT	−
F1551^•^	stomach	low	F	48	WT	Del557-560	WT	−
F1552^•^	stomach	low	M	68	WT	Dupl575-588	WT	−
F1553^•^	stomach	moderate	M	75	WT	Dupl578	WT	−
F1554^•^	stomach	low	F	84	WT	Dupl571-579	WT	−
F1555^•^	stomach	low	F	65	WT	V559D	WT	−
F1556^•^	stomach	low	M	83	WT	V559D	WT	−
F1512^▴^	small intestine	high	F	62	WT	V559A	WT	−
F1513^▴^	stomach	moderate	F	80	WT	V560D	WT	−
F1725^▪^	stomach	high	M	72	WT	567_569delinsC	WT	−
F1726^▪^	stomach	very low	M	76	WT	570_576del	WT	−
F1727^▪^	duodenum	moderate	F	42	WT	570_576del	WT	−
F1728^▪^	other	−	M	73	WT	557_558del	WT	−
F1729^▪^	other	−	M	88	WT	550_558del	WT	−
F1730^▪^	small intestine	high	M	49	WT	557_558del	WT	−
F1731^▪^	stomach	moderate	F	72	WT	557_558del	WT	−
F1733^▪^	−	−	M	72	WT	557_558del	WT	−
F1734^▪^	small intestine	moderate	F	72	WT	559_573del	WT	−
F1735^▪^	duodenum	moderate	F	73	WT	557_558del	WT	−
F1736^▪^	small intestine	moderate	M	39	WT	556_571del	WT	−
F1741^▪^	stomach	moderate	M	39	WT	567-576SdelinSCV	WT	−
F1744^▪^	−	−	M	−	WT	557_558del	WT	−
F1745^▪^	small intestine	high	F	72	WT	556-570delinsH	WT	−
F1746^▪^	small intestine	moderate	F	62	WT	536_572del	WT	−
F1747^▪^	small intestine	high	M	68	WT	560_576del	WT	−
F1748^▪^	rectum	very low	M	67	WT	550_558del	WT	−
F1749^▪^	small intestine	very low	F	40	WT	560_576del	WT	−
F1750^▪^	duodenum	moderate	F	59	WT	563_573delinsME	WT	−

GISTs included in mRNA^▴^ analysis, first^•^ and second^▪^ miRNA analyses.

* Risk of relapse was assessed using the Miettinen and Lasota classification which comprises the mitotic count, the maximal tumor diameter and the anatomic site.

Abbreviations: M, male; F, female; poly, polymorphism of *PDGFRA* exon 10; −, unknown data; Dupl, duplication.

**Figure 6:**
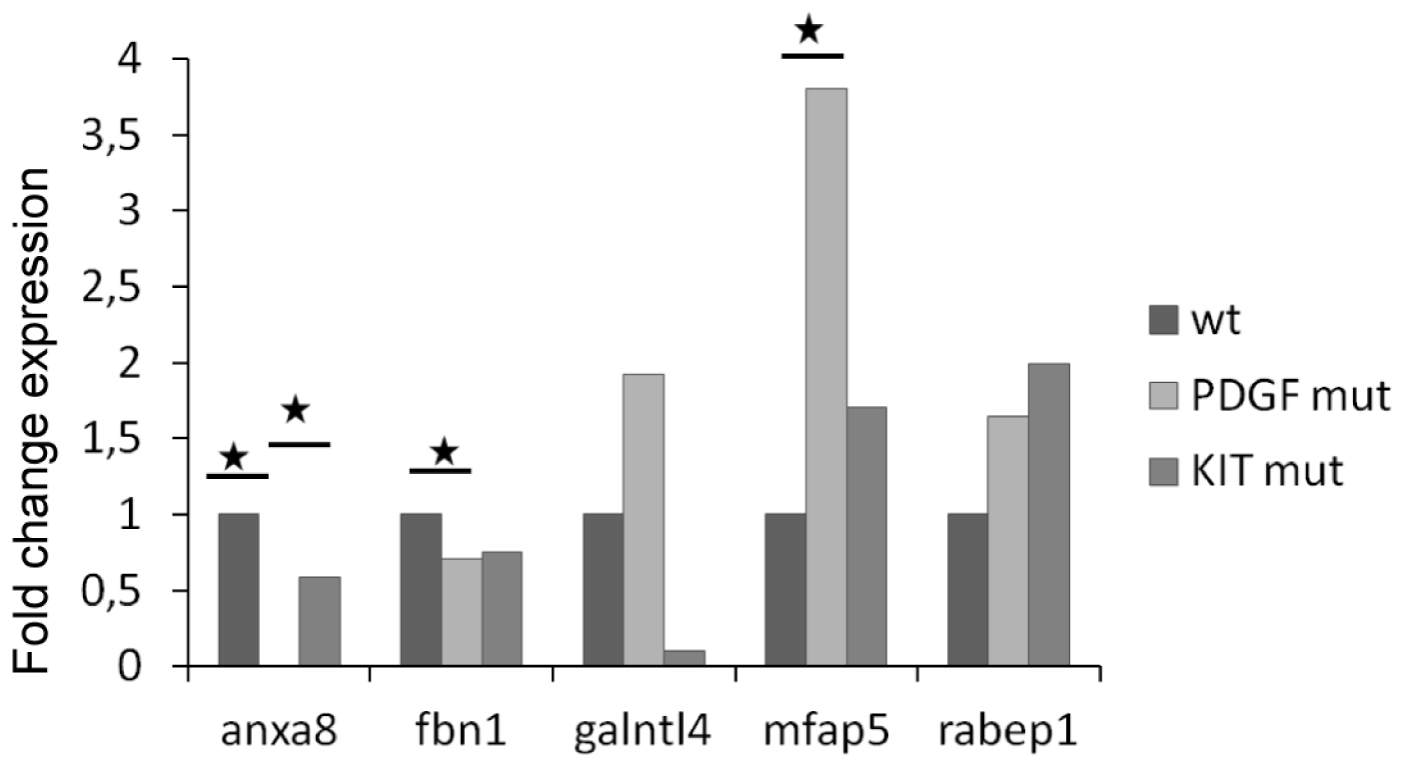
mRNA expressions of selected genes in GISTs according to their mutational status. mRNA expressions of five selected genes were analyzed in 16 GISTs: *Anxa8*, *Fbn1*, *Galntl4*, *Mfap5* and *Rabep1*. Among the 16 GISTs, 4 GISTs were WT *KIT* and *PDGFRA*, 5 harboured a *PDGFRA* mutation and 7 a *KIT* mutation. Results of mRNA expression levels in *PDGFRA* or *KIT* mutated GISTs are reported as N-fold differences in gene expression relative to the mRNA expression in WT GISTs. Mann Whitney test was used to assess significant difference.

For the mRNA analysis in GIST samples, we focused on ANXA8, FBN1, GALNTL4, MFAP5 and RABEP1. We observed the upregulation of MFAP5 in the mutated GISTs when compared to the WT tumors (Figure 6). This gene seemed particularly affected in *PDGFRA* mutated GIST (U test *p* = 0.02). Anxa8 was differentially expressed between WT and *PDGFRA* mutants (U test *p* = 0.01), and between *KIT* mutants and *PDGFRA* mutated tumors (U test *p* = 0.03). FBN1 was significantly less expressed in *PDGFRA* mutants than in WT tumors (U test *p* = 0.02). Interestingly, by separating exon 9 and exon 11 mutated tumors standard deviation for *GALNTL4* expression was reduced, so that a significant difference in its expression was found between *PDGFRA* mutants and *KIT* exon 9 mutated GIST (U test *p* = 0.03).

**Figure 7:**
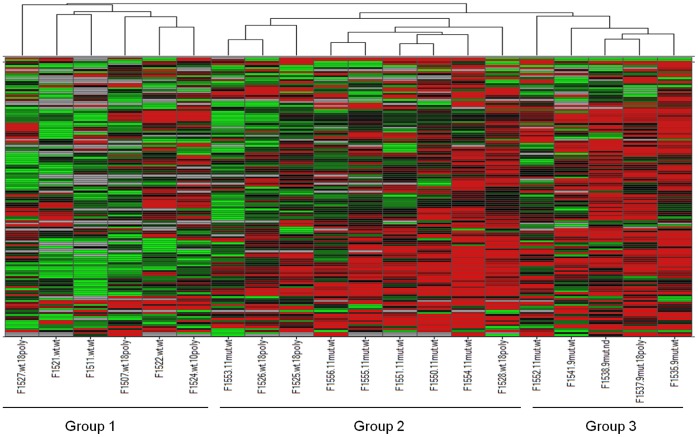
Unsupervised hierarchical clustering of miRNA expressions in 20 GIST samples. The four main groups of the dendogram are gathered by mutation type: group 1, WT *KIT* GIST; group 2, *KIT* exon 11 or *PRGFRA* exon 18 mutation; group 3, *KIT* exon 9 mutation and one tumor with *KIT* exon 11 mutation.

Twenty GISTs were included in the first miRNA analysis: three were WT, while the others harbored heterozygous mutations involving either *PDGFRA D842V* mutations (n = 6) or *KIT* (n = 11). Unsupervised hierarchical clusterings of these tumors were performed (Figure 7). It highlighted three distinct groups of tumors, mainly consistent with their mutational status. Group 1 was exclusively composed of WT *KIT* tumors; group 2 contained both *PDGFRA* and *KIT* exon 11 mutated GIST. *KIT* exon 9 mutated tumors were localized in group 3.

**Figure 8:**
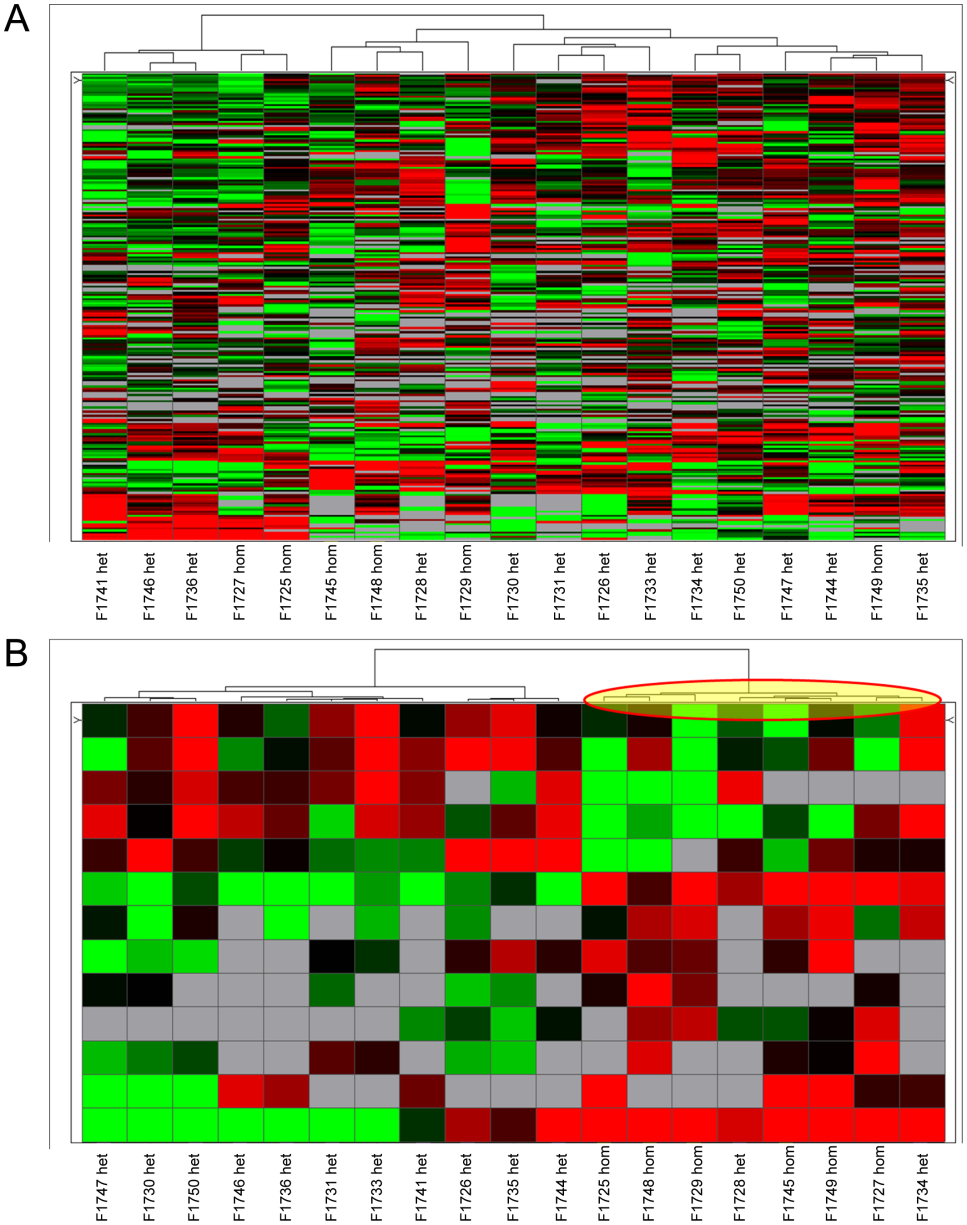
Hierarchical clustering of miRNA expressions in 19 GIST samples with exon 11 mutation (homozygous vs heterozygous). A/Unsupervised hierarchical clustering of miRNA expressions in 19 GIST samples. GIST samples were not classified according to their homozygous or heterozygous status in this unsupervised analysis. B/Supervised hierarchical clustering of GISTs using the 13 miRNA significantly deregulated (Anova test *p*<0.05). Homozygous and heterozygous tumors clustered separately except two heterozygous GISTs which were grouped with the homozygous.

In the second miRNA analysis, 19 GIST samples with homozygous or heterozygous exon 11 *KIT* mutation were included. Thirteen mRNA were specifically related to homozygous/heterozygous status of *KIT* mutation: miR-518f, miR-331, miR-628, miR-145, miR-139, miR-335, miR-526b, miR-190, miR-548c, miR-202, miR-339, miR-203, and miR-301b (Anova test *p*<0.05). MiR-222 was the only common miRNA found between the cell lines and GIST samples analysis but was not statistically significant. Six genes deregulated between cell lines are targeted by miR-548c, miR-145, miR-301b, and miR-628-5p: *Anxa8*, *Fbn1*, *Ptx3*, *Upp1*, and *Usp33*. Unsupervised hierarchical clusterings of these 19 tumors were performed and did not classify the tumors according to their homozygous/heterozygous status (Figure 8A). Supervised hierarchical clusterings were then performed using the 13 miRNA significantly deregulated between the homozygous and heterozygous tumors with Anova test. With this methodology, homozygous and heterozygous tumors clustered separately except two heterozygous GISTs which were grouped with the homozygous (Figure 8B). Using a 5% threshold for the p-values, the regression analyses and the Fisher's exact tests revealed no significant relation between the clinical data and the homozygous/heterozygous status (Table 3).

**Table 3:** Relationship between clinical data and the homozygous/heterozygous status.

Factors		Univariate logistic regression pvalue	Fisher’s exact test pvalue
Age (years)	<60	0,327	0,673
	≥60	0,657	
Gender	Female	0,327	0,661
	Male	0,593	
Tumor Size (cm)	<6	0,147	0,665
	≥6	0,973	
Mitosis (/5 mm2)	<2	0,083	1
	≥2	0,973	
Necrosis	No	0,637	
	<50	0,484	
	>50	0,995	
Risk of relapse	High	1	
	Moderate	0,184	
	Very Low	0,997	
Histology type	Epithelioid	0,997	
	Fusiform	0,997	
	Mixt	0,997	
	Non Gist	1	
	Pleomorphic	0,997	
Primitive location	duodenum	0,341	
	stomach	0,779	
	small intestine	0,779	
	jejunum-illeum	0,997	
	mesocolon	0,999	
	pelvis	0,999	
	perianal	0,999	
	rectum	0,999	
Kit Immunohistochemistry	+	1	
	++	0,501	
	+++	0,404	
	0	0,994	
Kit intracellular locations			
Golgi	No	0,079	0,635
	Yes	0,413	
Membrane	No	0,484	0,631
	Yes	0,481	
Cytoplasm	No	0,996	0,526
	Yes	0,996	

## Discussion

The discovery and the characterization of *KIT*/*PDGFRA* role in GISTs pathogenesis has led to a better identification of GIST among others gastrointestinal stromal tumors and to the development of targeted therapies in this disease. The KIT immunostaining, as well as the molecular analysis of the *KIT and PDGFRA* genes became major diagnostic tools [26]. The KIT inhibitor, imatinib mesylate, showed dramatic antitumor effects and is representing now the standard treatment in advanced GIST, as well as in adjuvant setting [9], [11]. Typically, GIST are heterozygous, but loss of the remaining WT *KIT* allele occurs in approximately 5% to 15% of tumors and homozygous GISTs seem to associate with a malignant behavior in 90% of cases [13], [14], [15]. This worsened prognosis could be explained by at least two opposite hypotheses. One the one hand, homozygous status with the loss of the wild-type allele may have a highly intrinsic oncogenic effect, possibly by loss of regulatory mechanisms of KIT. On the other hand, homozygous status may be only a marker of late-stage tumors that have undergone multiple genetic events without specific oncogenic effect.

These data led us to develop an adequate cellular model expressing hemizygous or heterozygous *KIT* mutation in the aim to better understand the molecular implications of the hemi/heterozygous status. Previous work on our NIH3T3 cellular model allowed us to characterize hemizygous human *KIT* mutants called D6 (Δ557–558) and D54 (Δ564–581) [19], as well as heterozygous ones [21]. In the present work, we completed these observations by analyzing these different cell lines, using high throughput tools and validation with GIST samples. Both mRNA and miRNA analyses demonstrated the close relationship between heterozygous mutants and WT samples, the distance with hemizygous *KIT* mutants and the activation of specific signaling pathways.

The supervised hierarchical clustering of miRNA of 19 GISTs with exon 11 mutation confirmed that homozygous and heterozygous GISTs tend to cluster separately, while it was not the case in the unsupervised hierarchical clustering. In contrast, the unsupervised hierarchical clustering of the more heterogeneous population of 20 GISTs, demonstrated a good segregation of samples according to the type of mutation (WT, *KIT* exon 11, 9 or *PDGFRA*). This suggests that, while the heterozygous/homozygous status have a real impact on the tumor expression profile, it may be weaker than the type of mutation.

Interestingly, while epidemiologic data [13], [14], [15], and mouse experiments reported clinic-pathologic and phenotypic differences between homozygous and heterozygous KIT activating mutations [27], [28], this study is among the rare in vitro studies evaluating the biological significance of that. Indeed, we recently shown in the same in vitro model that glycosylation, membrane expression and signal transduction of KIT are different in mutant and WT [19], and are also different in hemizygous and homozygous conditions [21].

We showed in the present study, that some genes were differentially expressed in hemizygous cell lines comparing to the cell lines containing the WT allele. The membrane protein RGS16 was found differentially down-regulated in hemizygous or heterozygous mutants when stimulated with SCF. In contrast, upon kinase inhibition, it was keep deregulated only in hemizygous mutants. Interestingly, RGS16, which is a member of the regulator of G protein family, was reported to negatively regulate the PI3K/AKT activity by competitively prevent the binding of p85α with the tyrosine kinase receptor adaptor Gab1 [29]. Consistently, we previously reported that AKT was less phosphorylated in hemizygous cell lines than in WT and heterozygous cell lines in presence of SCF, and that the inhibition of KIT membrane expression was associated with a major decrease of phosphorylated AKT in WT and heterozygous cell lines [21].

Furthermore most of the genes we found differentially regulated in hetero- homozygous conditions were already shown to be implicated in cancers, and several studies supported their biological significance. Loss of RGS16 was thus proposed to promote tumors by increasing proliferation and evasion from tyrosine kinase inhibitors treatment [29], [30]. The gene Mfap5, or Magp2, was also particularly upregulated in hemizygous mutants while heterozygous showed expression level very closed to the WT. It encodes a microfibril-associated glycoprotein implicated in Notch signaling pathway, and its overexpression has been reported as a poor prognostic biomarker in ovarian and head and neck cancers, probably through a proangiogenic effect [31], [32]. In GIST, the Notch pathway has been described recently as implicated in negative regulation of KIT expression, and high expression of *HES1 (hairy and hencer of split 1)*, one of the targeted genes of the Notch pathway, was associated with a better prognosis in patients with GIST [33]. Interestingly, Mfap5 was reported as able to promote angiogenesis by antagonizing Notch signaling pathways in endothelial cells [34]. Thus, the interactions between KIT, HES1 and Mfap5 may be implicated in GIST pathogenesis and malignancy.

Most of the 23 others genes differently regulated in WT cells, heterozygous and hemizygous mutants are involved in cancers. A first group is implicated in cell trafficking and endocytosis, such as the golgi protein Galntl4, Rabep1 (or rabaptin), implicated in endocytosis [35], [36], annexin A8 (Anxa 8) which directly regulates organization and function of the late endosome [37], [38], and the deubiquitinase USP33 which inhibits the lysosomal trafficking [39], [40]. A second group of genes encodes for proteins which are implicated in cell-to-cell interactions and cell-matrix interactions: Farp2, Fbn1, Ncam1 and Thbs1. Fbn1 encodes the extracellular matrix protein fibrillin-1, and its mutation results in the dominant connective tissue disease Marfan syndrome. Quantitative methylation analyses have recently reported that the promoter of Fbn1 was hypermethylated in colorectal and prostate cancers in comparison to normal tissues, suggesting that this gene could play a role in oncogenesis [41], [42]. Slit3 is a secreted protein whose family is known to regulate migration of neural cells, and its downregulation seems implicate in progression of various cancers [43], [44]. A third group of proteins is involved in cell signaling or gene regulation: Wisp 1, Wisp2, Nf1, Ulk1, Runx1t1. Wisp1 and Wisp2 are members of the Wnt inducible signalling pathway (WISP) protein subfamily and have been described as deregulated in several cancers [45], [46], [47], [48], [49]. Activation of Wnt signaling results in the inhibition of a degradation complex which targets β-catenin for proteosomal degradation. The accumulation of β-catenin in the cytoplasm then its translocation to the nucleus and its binding to T-cell specific transcription factor/lymphoid-enhancer binding factor (TCF/LEF) complex led to the modulation of target genes implicated in cell cycle regulation [50]. Recently, upregulation of the Wnt signaling through autocrine activation has been reported in sarcoma cell lines and in human sarcomas [51]. In these tumors, Wnt activation resulted in cell proliferation via upregulation of the TCF/β-catenin target gene *CDC25A* which plays a nonredondant role in regulating cell cycle [51]. Nf1 which is a negative regulator of the ras signal transduction pathway, mutated in neurofibromatosis type 1, also confers an increased risk of developing WT GISTs [52]. Ulk1, which plays an essential role in autophagosome formation through activation or regulation of the AMP activated protein kinase and mTOR, is overexpressed in esophagus squamous cell carcinoma [53]. Runx1t1 encodes a member of the myeloid translocation gene family and recruit a range of corepressors to facilitate transcriptional repression. Chromosomal translocations involving *Runx1t1* are well-documented and have been associated with several types of leukemia [54].

The presence or absence of the two tyrosine residues Tyr568 and Tyr570, may be associated with various effects in KIT regulation and/or signaling pathways activations. Phosphorylated Tyr568 serves as a docking site for the Src-like kinase Fyn which activate the Rac1/JNK pathway, for the adapter protein Shc which activate the Ras/MAP kinase pathway, and for the adapter protein APS and the ubiquitin E3 ligase Cbl which may play a role in the degradation of KIT [55], [56], [57], [58], [59]. The Csk homologous kinase (CHK) and the Src kinase Lyn bind to phosphorylated Tyr568 and Tyr570 [60], [61]. SHP-1 and SHP-2 tyrosine phosphatases bind to non-phosphorylated Tyr570 and Tyr568, respectively, and may negatively regulate KIT activation [62]. Consequently, a deletion of both Tyr568 and Tyr570 could disturb the autoinhibition conformation of exon 11 but also alter some protein-protein interactions and signaling pathway activations. Consistently, we demonstrated different mRNA and miRNA expression profiles in D6 and D54 cells. Thus, the loss of both *KIT* exon 11 tyrosine residues was associated with specific modifications in gene expression. For example, Wisp2 was overexpressed in D54 cells in comparison to D6 cells whereas Wisp1 was expressed at similar level in both cell lines. Others genes differentially expressed between D6 and D54 cells (more than 3 times) in all conditions (without SCF, with SCF, with IM) were: Galntl4, Prelp, Ptx3 were overexpressed in D54 cells whereas Gpr149, Ifi47, Ncam1, Upp1 were overexpressed in D6 cells.

No clinical data was correlated with the homozygous/heterozygous status but, consistently with previous publications, there was a tendency for a correlation between the Golgi pattern in immunohistochemistry and the homozygous status. In our cell models, mutant KIT was mainly retained within Golgi and endoplasmic reticulum compartments whereas WT KIT was expressed at the plasma membrane [19]. Moreover, in GIST samples, the Golgi staining was significantly more frequent in GISTs with homozygous mutations than in heterozygous [63].

In tumor samples gene expression analysis showed no significant variation between WT and heterozygous *KIT* mutated tumors, with a high inner variability in *ANXA8*, *FBN1*, *GALNTL4*, *MFAP5* and *RABEP1* expression, which was partly reduced by separating exon 9 and exon 11 mutated tumors. While this absence of variation is globally quite consistent with the closed profiles we observed between WT and heterozygous cell lines, the inner variability of mutation type in the *KIT* mutant group might also participate to this result. For the future we will need to significantly increase the number of the cohort to validate our hypothesis. On the contrary, our results on the 5 *PDGFRA* mutated tumors were very stable. The striking stability of our gene expression results on *PDGFRA* mutated tumors could be explained by the D842V mutation found on 4/5 samples. The fifth tumor exhibited an exon 10 polymorphism and had expression values in the range of exon 18 mutated tumors. The response to targeted therapies, such as imatinib and sunitinib, is also dependent of the type of mutation [11], [12], [64]. Interestingly, miRNA and mRNA profiles also suggested that the two mutations do not respond in the same way to imatinib treatment as their profile that clustered initially were then segregated under treatment (Figures 3A *vs* 3B).

In conclusion, our results shown that heterozygous/hemizygous status, as well as the presence or not of the two *KIT* exon 11 tyrosine residues, were associated with specific modifications in gene expression of both mRNA and miRNA. These results suggest that deletion of the WT allele of *KIT* or deletion of the two tyrosines Tyr568 and Tyr570 may be associated with specific biological effects. Interestingly, most of the gene found frequently deregulated between cell lines encoded proteins which have been reported as deregulated in cancer. These results demonstrate the relevance of our cellular model and may allow to better understand other molecular events associated with GIST pathogenesis and malignancy. We have also clearly shown that both mRNA and miRNA profiling allowed the gathering of some tumors according to their mutational status, giving complementary results. A more complete mRNA and miRNA screening should be performed and followed by an integrative analysis of the results, including clinical follow-up information for each patient. Such search strategy could allow to identify new molecular targets and/or signaling pathways driven GIST pathogenesis, and to define new GIST subgroups.
